# Double Sublaminal Band Passage Technique for Spinal Deformity Correction

**DOI:** 10.7759/cureus.22719

**Published:** 2022-02-28

**Authors:** Blake K Montgomery, Sreeharsha V Nandyala, Craig M Birch, Grant Hogue

**Affiliations:** 1 Department of Orthopaedic Surgery, Boston Children’s Hospital, Boston, USA

**Keywords:** spine deformity surgery, surgical technique, spine, double passage, sublaminal band

## Abstract

Sublaminar band fixation is a reliable way to anchor spinal rods to the vertebral column. This technique is especially useful when the anatomy precludes safe pedicle screw placement. Sublaminar bands allow for deformity correction and stabilization of the spine. One of the disadvantages of using the sublaminar band technique is the risk for neurologic injury during the passage of the band between the dura and lamina. In this article, we describe a new technique for passing sublaminar bands, i.e., the double sublaminar band passage technique. This technique decreases the number of passes against the dura, thereby decreasing the opportunity for neural injury. In addition, we present an illustrative case of an 11-year-old female with neuromuscular scoliosis who underwent a posterior spinal instrumented fusion with a hybrid screw and sublaminar band construct.

## Introduction

Polyester sublaminar band (SB) fixation has gained considerable popularity as a construct anchor for the correction of pediatric spinal deformity [1-3]. The SB passage technique has been demonstrated to be safe and effective in the thoracolumbar spine [4-6]. The neurologic complication rate is 0.8%, similar to that of other anchor fixation constructs [4].

SBs are particularly effective at restoring thoracic kyphosis by reducing the vertebrae to the rod with a sequential reduction technique. Hybrid constructs, which include both thoracic pedicle screw fixation along with SBs are associated with improved thoracic kyphosis compared to traditional all-pedicle screw constructs for the treatment of thoracic adolescent idiopathic scoliosis (AIS) [7,8]. Polyester SBs have been utilized in both AIS and neuromuscular scoliosis settings [9]. They can be particularly helpful at spinal levels where dystrophic pedicles or poor bone quality precludes safe pedicle screw placement [6]. Contraindications to SB utilization include lack of a lamina such as in spina bifida or in patients who have a history of previous intra-canal procedures such as dorsal root rhizotomies.

SB passage requires creating a channel under the lamina, thereby exposing the dura and spinal cord to injury. In most situations, SBs are often paired at each intended spinal level and require passage under the cortical lamina twice if each band is passed separately. To mitigate injury to the dura and spinal cord, we present a dual-band passage technique whereby two bands can be passed with only one pass under the lamina.

## Technical report

The patient is placed prone on a well-padded Jackson frame (Orthopedic Systems Inc., Union City, USA). Neuromonitoring should be utilized for all cases. After the appropriate surgical spinal levels are identified, an incision is made, and dissection is carried out in a standard subperiosteal fashion. Care is taken not to violate the interlaminar spaces during dissection. The designated levels for SB anchors are determined based on the need for restoring kyphosis, inability to place pedicle screws safely, or surgeon preference.

In the thoracic spine, shingling of the thoracic spinous process and lamina of the cephalad level necessitate removing the caudal end of the lamina. This may be planned in the apex of the curve where Ponte osteotomies are already planned. Care is taken to identify the ligamentum flavum at the caudal end of each lamina, where it is the thickest. Kerrison rongeur is utilized to remove the ligamentum flavum. A Woodson is utilized to create a passage from underneath the caudal end of the lamina to the cephalad end.

**Figure 1:**
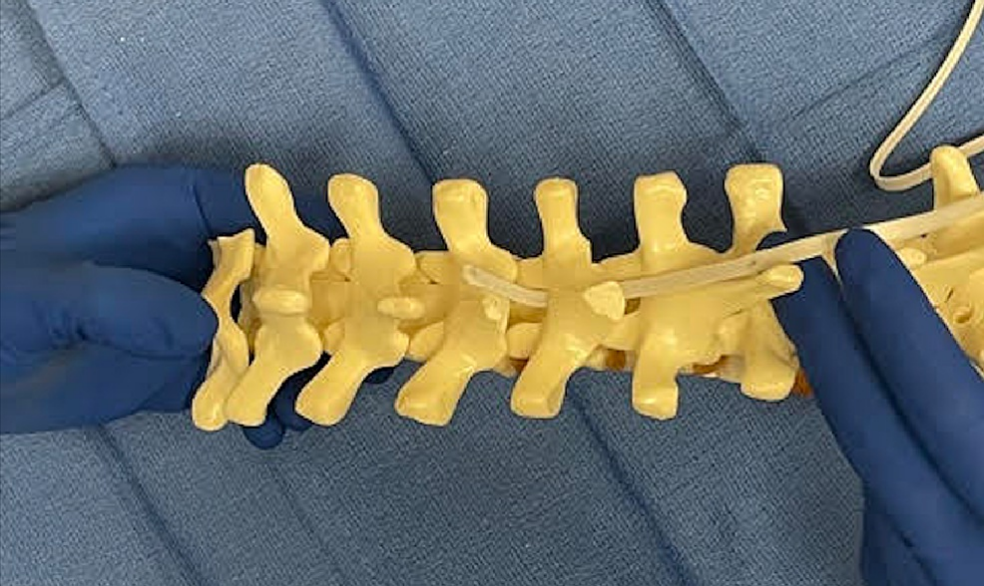
Demonstration of the passage of the first SB on a spine model. The spinous process and caudal aspect of the lamina have been removed.

**Figure 2:**
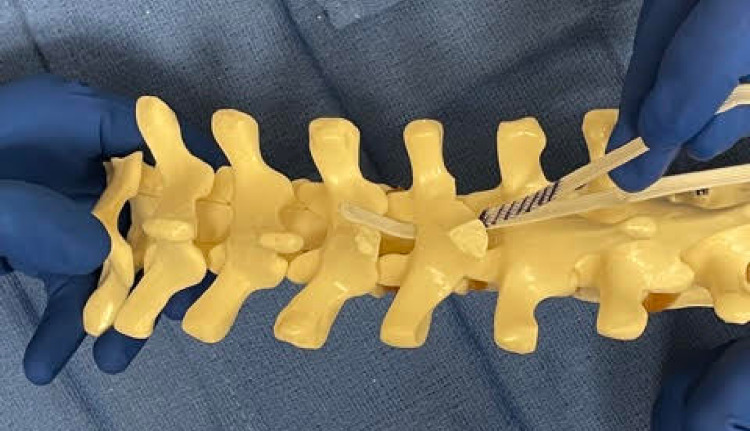
The second band (purple striped) is passed between the first band and the lamina. The first band acts as a shield to help protect the neural structures.

**Figure 3:**
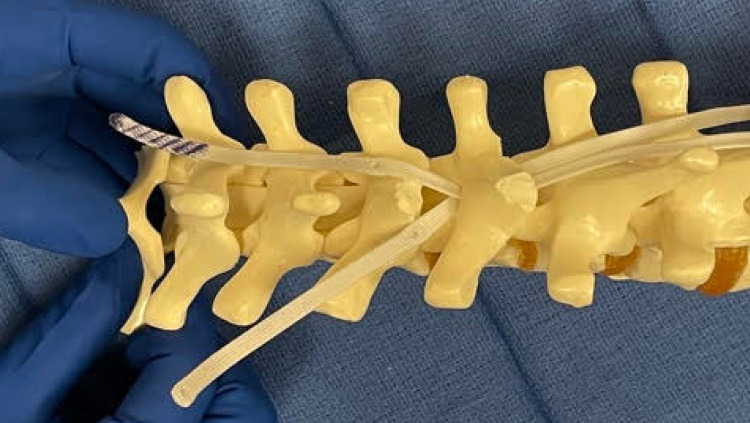
Both bands are pulled cranially and adjusted to their appropriate position.

**Figure 4:**
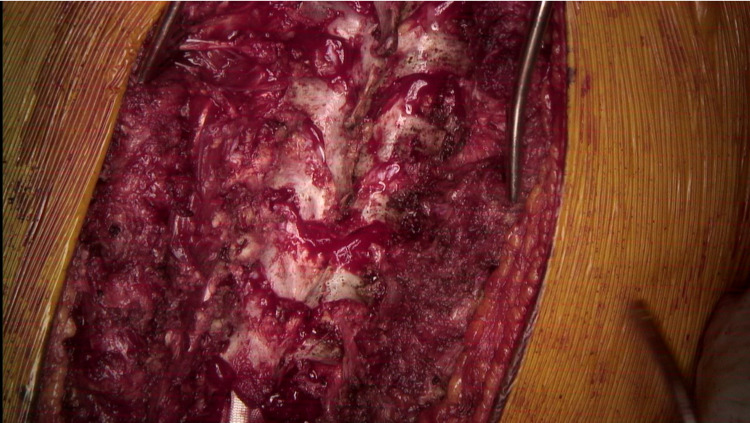
Intraoperative image of the posterior spine during posterior spinal instrumented fusion with the utilization of the double sublaminar band passage technique demonstrating central laminectomy and flavectomy in preparation for SB passage.

**Figure 5:**
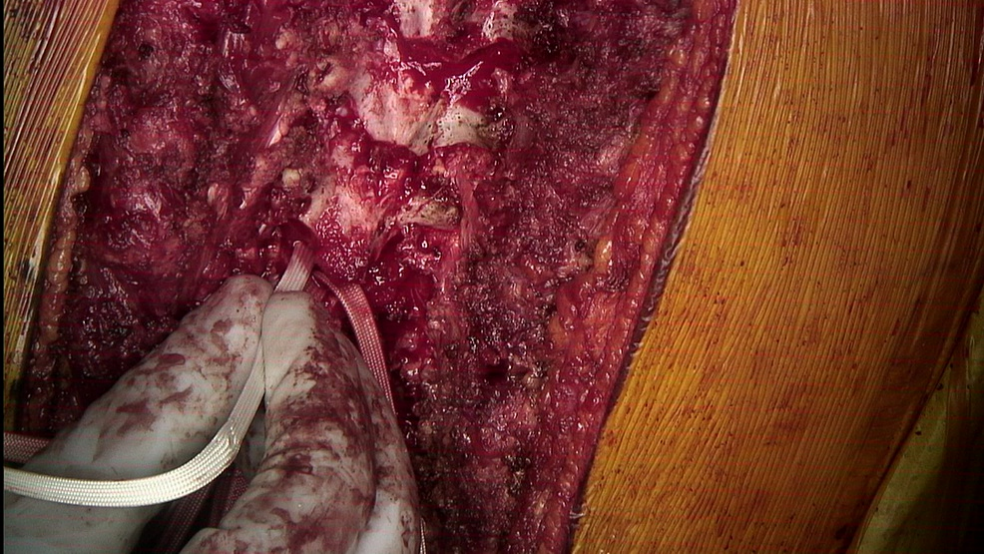
The first SB passage from caudal to cranial is done being careful to avoid pressing on the dura and spinal cord.

**Figure 6:**
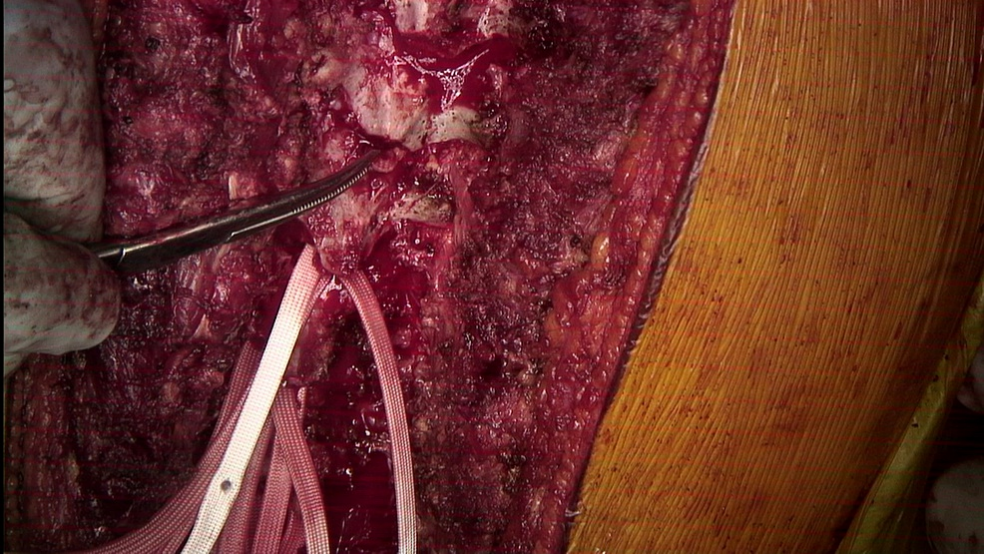
A hemostat is used to pull the first SB cranially.

**Figure 7:**
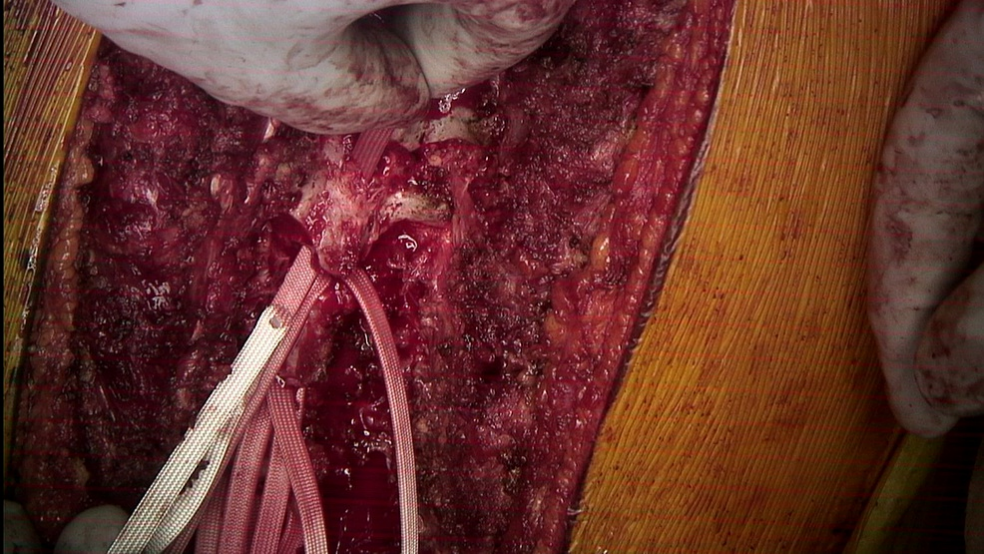
SB after it has been passed beyond the lamina.

**Figure 8:**
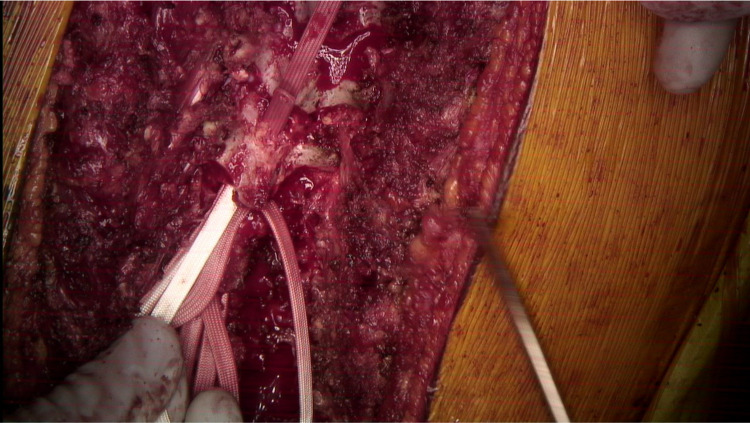
A second SB is passed from caudal to cranial posterior to the first band. The first band acts as a sled and provides a layer of protection from the dura and spinal cord.

After creating a central passage, the leading end of polyester SB is contoured to the appropriate curvature of the lamina (Figures 1-8). Care must be taken not to over contour the leading edge to prevent the heel/apex of the curve from pressing against the spinal cord during the passage. The leading edge of the sublaminar band is gently passed through the central passage. Care is taken not to press down during the passage. A hemostat or needle driver is utilized to pull up the leading edge after passage across the lamina.

A second SB is contoured similarly. The leading edge of the second SB is then passed under the lamina and over the previously passed SB. Once again, care is taken not to push down. The first band acts like a “sled” to facilitate the second band passage without direct contact with the dura. After the second band is passed, a needle driver is utilized to pull up the second band and to adjust both bands to their final positions.

Illustrative case

**Figure 9:**
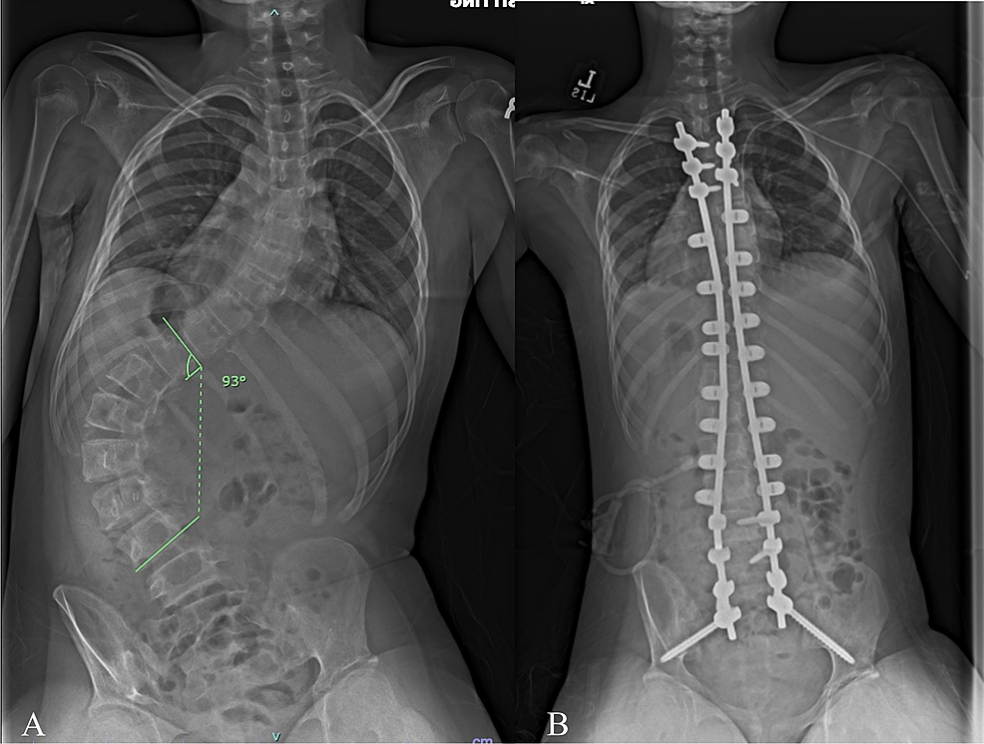
A) Preoperative and B) postoperative sitting PA views. The preoperative x-ray demonstrates a 93 deg left-sided thoracolumbar curve with 11 degrees of pelvic obliquity. The postoperative x-ray demonstrated T3 to pelvis instrumented fusion with the utilization of multiple sublaminar bands. The thoracolumbar curve improved to 9 degrees.

**Figure 10:**
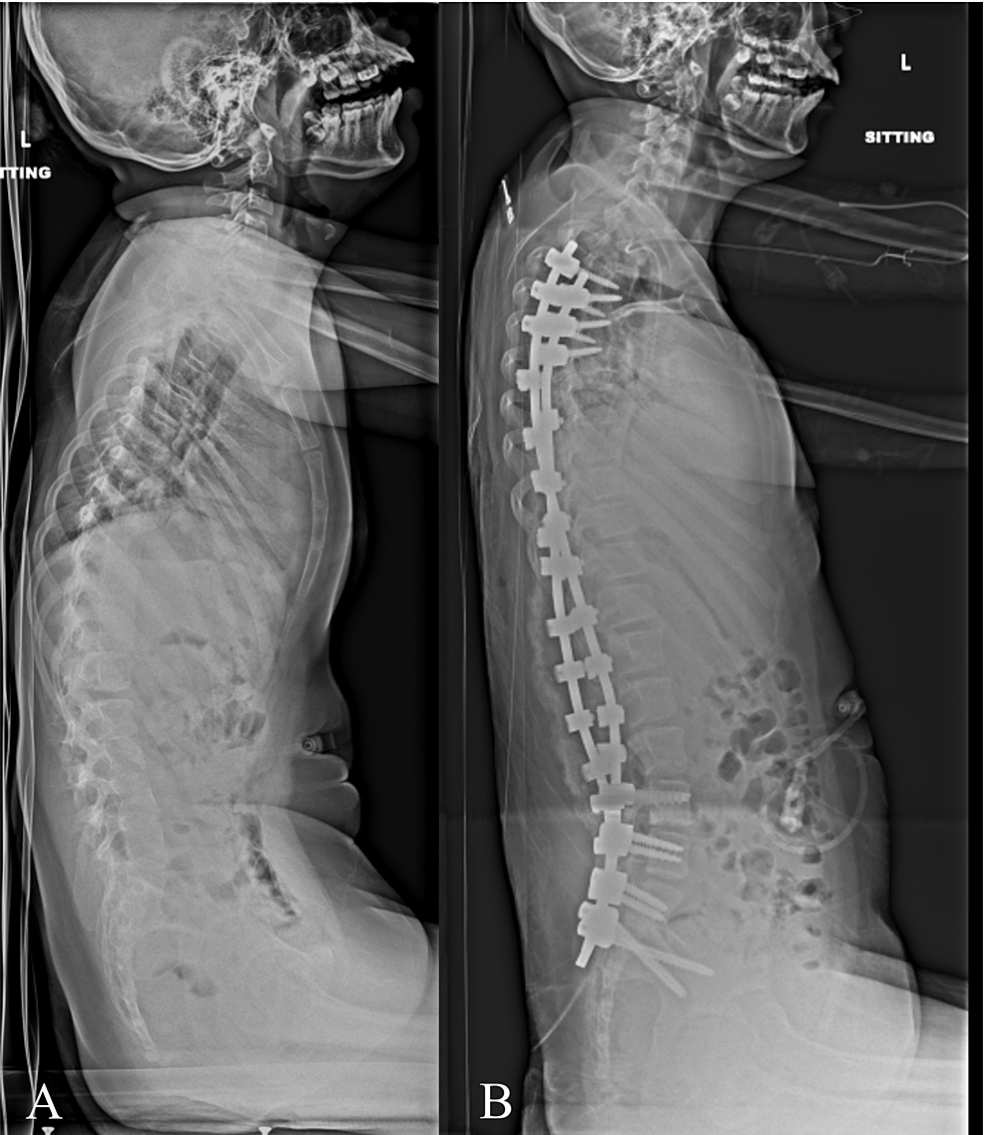
A) Preoperative and B) postoperative sitting lateral x-ray. The preoperative x-ray demonstrates loss of lumbar lordosis (2 degrees), thoracolumbar kyphosis (13 degrees), and positive sagittal balance. The T5-T12 thoracic kyphosis measured 30 degrees. The postoperative x-ray demonstrates T3- pelvis instrumented fusion with the utilization of multiple sublaminal bands with improvement in sagittal balance (T5-T12 thoracic kyphosis 27 degrees, lumbar lordosis 27 degrees, thoracolumbar angle 2 degrees).

**Figure 11:**
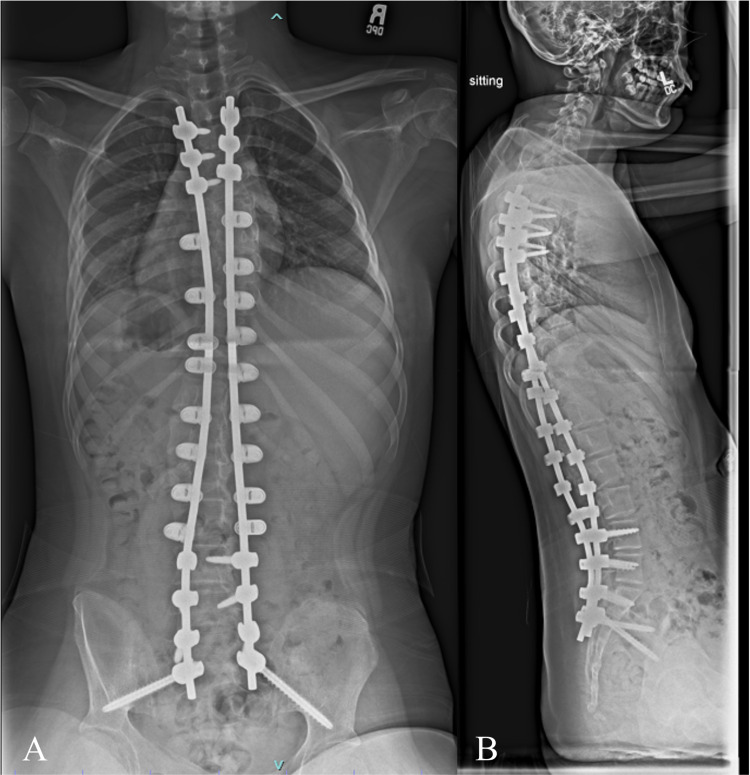
A) PA and B) lateral x-rays from one-year post-operative visit demonstrating fusion mass posteriorly, maintained deformity correction, and intact implants.

An 11 year female with spastic quadriplegic cerebral palsy, gross motor function classification scale level four, and progressive neuromuscular scoliosis was referred to the spine surgery clinic. Physical examination revealed a left thoracolumbar prominence with waist asymmetry and right-sided waist indention between the ribs and the iliac wing without much space in between. X-rays demonstrated a 93 deg left-sided thoracolumbar curve and 11 degrees of pelvic obliquity (Figures 9, 10). She underwent T3-pelvis posterior spinal instrumented fusion with the utilization of the double SB passage technique. No intraoperative monitoring changes were encountered. The patients' exam was unchanged postoperatively. One year follow-up visit demonstrated maintained correction and intact implants (Figure 11). 

## Discussion

Anchor point fixation for AIS and neuromuscular (NM) scoliosis is of critical importance for proper spinal deformity correction and stabilization. SB utilization enables the surgeon to obtain an anchor point even when anatomy precludes safe pedicle screw placement.

SB passage puts the underlying spinal cord at risk. Even with meticulous surgical technique, a large retrospective series demonstrated the rate of neural injury of 0.8% (three patients out of 378 operative procedures) [4]. Every time an instrument is passed against the dura, there is a risk for neural injury. A higher number of passes under the lamina means an increased opportunity for neural injury. Often two SBs are passed at the same vertebral level. The traditional technique of SB passage necessitates two separate passes against the dura if two bands are utilized at the same level. In contrast, our technique requires only one pass against the dura. This technique decreases the opportunity for neural injury compared to the traditional technique.

Our proposed technique has limitations. First, we have not experienced any neurological injuries using this technique; however, since the baseline rate of neurological injury is low, hundreds of patients would be required to detect a lower neurological injury rate compared to the traditional SB technique.

## Conclusions

In conclusion, sublaminar bands are reliable implants, allowing for rods to be anchored to the spine for deformity correction and stabilization. The double sublaminar passage technique minimizes the opportunity for neural injury while allowing two sublaminar bands to be utilized at one level. We recommend consideration of this technique when two sublaminar bands are applied at a single level.
